# Complete Genome Sequence of Nisin-Producing Lactococcus lactis subsp. *lactis* N8

**DOI:** 10.1128/MRA.01147-20

**Published:** 2021-01-07

**Authors:** Xing Wan, Timo M. Takala, Mingqiang Qiao, Per E. J. Saris

**Affiliations:** a Department of Microbiology, Faculty of Agriculture and Forestry, University of Helsinki, Helsinki, Finland; b Key Laboratory of Molecular Microbiology and Technology, Ministry of Education, College of Life Sciences, Nankai University, Tianjin, China; University of Maryland School of Medicine

## Abstract

We report here the genome sequence of Lactococcus lactis subsp. lactis N8, a nisin producer isolated in the 1960s from a dairy product in Finland. The genome consists of a 2.42-Mb chromosome and two plasmids of 80.3 and 71.3 kb.

## Abstract (precis)

We report here the genome sequence of Lactococcus lactis N8, a nisin producer isolated in the 1960s from a dairy product in Finland. The genome consists of a 2.42-Mb chromosome and two plasmids of 80.3 and 71.3 kb.

## ANNOUNCEMENT

Lactococcus lactis is an important lactic acid bacterium in the dairy industry (1). Some L. lactis strains produce the antimicrobial peptide nisin, which is active against many foodborne pathogens (2, 3). L. lactis subsp. *lactis* N8 is a nisin Z producer isolated in the 1960s from a high-quality dairy product by the Finnish dairy company Valio Ltd. (4). We received strain N8 from Valio Ltd. in November 1990, and since then, we have conducted various studies to understand nisin biosynthesis (5–9) and to develop biotechnology tools (10–15).

Genomic DNA (gDNA) was extracted from a 2-ml culture of L. lactis N8 grown overnight at 30°C in M17 broth supplemented with 0.5% glucose using a MagAttract high-molecular-weight (HMW) DNA kit (Qiagen, Hilden, Germany) according to the supplier’s protocol. A DNA template prep kit, no. 2.0 (Pacific Biosciences, Menlo Park, CA, USA), was used to generate 3- to 10-kb fragments, and DNA/polymerase binding kit P6 (Pacific Biosciences) was used to generate a DNA polymerase/template library complex. The library was sequenced on a PacBio RS II sequencer, generating 103,204 reads (*N*_50_, 11,879 bp). Reads were *de novo* assembled using the RS hierarchical genome assembly process (HGAP) version 3.0 implemented in SMRTportal 2.3 (Pacific Biosciences).

Aliquots of gDNA were also used to generate a library with a Nextera XT kit and sequenced on the Illumina MiSeq platform using a 300-bp paired-end strategy with MiSeq reagent kit v3 (Illumina, Inc., San Diego, CA, USA), according to the manufacturer’s instruction. The Illumina reads were trimmed with cutadapt (v1.14; minimum length, 50 bp; quality cutoff, 25) (16), resulting in 418,075 R1 plus 418,075 R2 reads for mapping by Burrows-Wheeler Aligner (17) and for polishing the draft assembly by Pilon (18).

Complete genome sequences were circularized using a genome assembly program (Gap4, Staden package) (19), and overlapping ends were removed manually. Default parameters were used for all software unless otherwise noted.

The assembly generated three circular contigs, representing one chromosome and two plasmids, with sizes of 2,421,567 bp (506× PacBio coverage, 85× Illumina coverage), 80,301 bp (pLLN8-1; 178× PacBio coverage, 136× Illumina coverage), and 71,261 bp (pLLN8-2; 178× PacBio coverage, 145× Illumina coverage), respectively. The closed genome was rotated to start at the *dnaA* gene. Like other L. lactis subsp. *lactis* strains, strain N8 exhibits a chromosomal G+C content of 35.1%. Its plasmids, pLLN8-1 and pLLN8-2, have G+C contents of 35.1% and 33.7%, respectively.

The genomic sequences were uploaded to NCBI and automatically annotated with NCBI Prokaryotic Genome Annotation Pipeline (PGAP) version 4.12 (20), which predicted a total of 2,521 gene sequences, including 2,434 coding sequences, 19 rRNAs, 64 tRNAs, and 4 noncoding RNAs. As expected, the genes *nisZBTCIPRKFEG*, which are needed for nisin biosynthesis, regulation, and immunity, were located in the nisin-sucrose transposon Tn*5481* in the chromosome. Interestingly, we also found a chromosomal gene cluster (>40 kb) for a nonribosomal peptide synthetase/polyketide synthase system.

### Data availability.

The complete genomic sequence of L. lactis subsp. lactis N8, including its plasmids, pLLN8-1 and pLLN8-2, has been deposited in GenBank and is available under the accession numbers CP059049, CP059050, and CP059051, respectively. Raw reads from PacBio and Illumina sequencing were uploaded to the Sequence Read Archive (SRA) under the accession numbers SRR12756349 and SRR13170739, respectively.
